# Translational Informatics Driven Drug Repositioning for Neurodegenerative Disease

**DOI:** 10.2174/011570159X327908241121062335

**Published:** 2025-02-06

**Authors:** Xin Zheng, Jing Chen, Yuxin Zhang, Shanshan Hu, Cheng Bi, Rajeev K. Singla, Mohammad Amjad Kamal, Katsuhisa Horimoto, Bairong Shen

**Affiliations:** 1 Institutes for Systems Genetics, Frontiers Science Center for Disease-related Molecular Network, West China Hospital, Sichuan University, Chengdu, Sichuan 610041, China;; 2 The School of Science, Kangda College of Nanjing Medical University, Lianyungang, Jiangsu 222000, China;; 3 Molecular Profiling Research Center for Drug Discovery, National Institute of Advanced Industrial Science and Technology, Tokyo, Japan

**Keywords:** Drug Repositioning, drug repurposing, neurodegenerative diseases, translational informatics, artificial intelligence, model, algorithm, database

## Abstract

Neurodegenerative diseases represent a prevalent category of age-associated diseases. As human lifespans extend and societies become increasingly aged, neurodegenerative diseases pose a growing threat to public health. The lack of effective therapeutic drugs for both common and rare neurodegenerative diseases amplifies the medical challenges they present. Current treatments for these diseases primarily offer symptomatic relief rather than a cure, underscoring the pressing need to develop efficacious therapeutic interventions. Drug repositioning, an innovative and data-driven approach to research and development, proposes the re-evaluation of existing drugs for potential application in new therapeutic areas. Fueled by rapid advancements in artificial intelligence and the burgeoning accumulation of medical data, drug repositioning has emerged as a promising pathway for drug discovery. This review comprehensively examines drug repositioning for neurodegenerative diseases through the lens of translational informatics, encompassing data sources, computational models, and clinical applications. Initially, we systematized drug repositioning-related databases and online platforms, focusing on data resource management and standardization. Subsequently, we classify computational models for drug repositioning from the perspectives of drug-drug, drug-target, and drug-disease interactions into categories such as machine learning, deep learning, and network-based approaches. Lastly, we highlight computational models presently utilized in neurodegenerative disease research and identify databases that hold potential for future drug repositioning efforts. In the artificial intelligence era, drug repositioning, as a data-driven strategy, offers a promising avenue for developing treatments suited to the complex and multifaceted nature of neurodegenerative diseases. These advancements could furnish patients with more rapid, cost-effective therapeutic options.

## 1 INTRODUCTION

Neurodegenerative diseases (NDDs) form a complex disease marked by neuronal damage and loss, progressively worsening over time [1]. Alzheimer's disease (AD), affecting approximately 38.5 million people globally, leads as the most prevalent NDD, followed by Parkinson's disease (PD), which impacts around 8.5 million individuals worldwide. The rise in life expectancy and an aging population present increasing challenges for NDDs, including growing prevalence, expanding patient populations, and rising healthcare demands. Current treatments for NDDs, which cannot cure but only alleviate symptoms, underscore the challenge of developing effective therapies [2]. Moreover, rare diseases like Huntington's disease (HD) and Amyotrophic Lateral Sclerosis (ALS) lack curative treatments [3].

Due to the unpredictable adverse reactions associated with new structural drugs, new drug development remains a significant challenge. Artificial intelligence (AI) allows drug development to be improved and accelerated through digital technology. A large volume of biomedical data generated by high-throughput technology lays the foundation for comprehensive analysis [4]. As a data-driven approach, drug repositioning (DR) is increasingly becoming a focus of attention [5, 6]. Compared with conventional new drug development, DR has the advantage of bypassing traditional new drug development such as preclinical experiments, safety reviews, clinical trials, and post-marketing safety monitoring. In addition to shortening the development cycle, DR has the advantage of reducing failure rates. Most drugs involved in DR have been in clinical use for a long time and have a large base of patient samples. Therefore, these drugs' safety and potential risks are well understood [7, 8]. Several successful cases have entered clinical practice on this basis. The AD drug Galantamine was initially used to treat polio in Eastern Europe during the 1950s [9]. Aspirin, which originally relieved pain and reduced fever, has been repurposed to treat heart disease, stroke, and thrombosis. Sildenafil citrate invented for angina now treats erectile dysfunction [8]. Thalidomide initially a sedative has been repositioned to treat erythema nodosum leprosum and multiple myeloma. Zidovudine originally conceived as a chemotherapy drug became the first FDA-approved antiretroviral drug [10]. Minoxidil used for high blood pressure has been approved for treating hair loss [11]. The new uses of drugs may also extend to related diseases. Bevacizumab from treating colon cancer to other solid cancers [12]. The melanoma drug pembrolizumab is being repurposed for 14 types of cancer [13]. Olaparib for ovarian and breast cancer now treats pancreatic cancer [14]. With the update of various computational technologies and the accumulation of a large amount of high-throughput data such as omics data, protein structure, and phenotype, data-driven DR can more accurately mine the multiple potential uses of existing drugs. The sudden outbreak of the COVID-19 epidemic led to insufficient time for new drug development [15]. DR provided new “old drugs” solutions such as Lopinavir/Ritonavir, Remdesivir, Azithromycin, and Favipiravir [16, 17]. DR demonstrated important value in a limited time and provided a more diverse choice for future clinical practice.

Although the development of AI and the upgrade of sophisticated technologies have significantly enhanced the ability to acquire and process data, the transformation of medical and health-related data into data-driven DR applications still faces considerable challenges [18]. Firstly, biomedical data is often fragmented with substantial differences in format, standards, and quality [19]. Secondly, the abundance of data leads to interference with effective data by noise and bias. In addition, data privacy and security in hospitals may limit data sharing and open access [20]. Therefore, there is an urgent need for new approaches that can facilitate the translation of health management data into clinical applications.

Translational informatics bridges research and clinical practice across disciplines. It primarily involves a process from collecting and parsing data to creating and validating models and ultimately applying them in clinical practice [21, 22]. Firstly, data collection and interpretation form the foundation of translational informatics. It involves the cleansing, standardization, integration, and feature extraction of data from basic experimental and clinical practice. Translational informatics encompasses data at its core. With data research progressing, transformations occur. Applying data across chemical information, biological information, medical information, and health information supports precision medicine [23, 24]. Secondly, the construction and validation of models serve as the tools in translational informatics. Using algorithms based on data features to make predictions or classifications and validating the model to ensure its generalization ability. Lastly, the application in clinical practice represents the objective of translational informatics. Models built from basic experimental and clinical trial data are used to predict the progression of diseases and the efficacy of new drugs [25, 26]. Thus, translational informatics integrates data and model research at four levels. DR driven by translational informatics represents a fresh paradigm. In the era of AI, we can construct more precise studies on DR and realize the vision of personalized medicine.

As shown in Fig. (**1**), this review aims to comprehensively examine drug repositioning for NDDs through translational informatics. It organizes databases, classifies computational models, and highlights current research while identifying potential databases for future efforts. Ultimately, it underscores drug repositioning as a promising, data-driven strategy for developing treatments, offering NDD patients faster and more precise options.

## 2 DATA RESOURCES FOR DR STUDY

Leveraging data resources and promoting standardization are pivotal for advancing DR. This effort requires acquiring broad, quality-controlled datasets. Data standardization is important to ensure data consistency, comparability, and communication across platforms and researchers [26, 27]. Research primarily focuses on organizing diverse data, developing disease-specific databases, sorting data based on drug and disease similarities for predictions, and standardizing data. These endeavors offer structured data and valuable knowledge for mining and novel discoveries in the field of DR. Table **1** provides a comprehensive list of the majority of databases in this area.

Integrating genes, targets, phenotypes, and drug data from multiple sources into a single platform deepens the exploration of their relationships. Chen *et al*. integrated eight drug databases to create the validated drug-target database HCDT [28]. Gonzalez-Cavazos *et al*. developed DrugMechDB, a knowledge graph-based database that details drug mechanisms, providing a benchmark dataset for computational model evaluation and training [29]. Yosef Masoudi-Sobhanzadeh used DrugBank and KEGG data to build DrugR+, enabling single and combination DR to discover optimal synergistic drug combinations [30].

Disease-specific databases accelerate personalized clinical treatments. Zhu *et al*. used ontology language and cheminformatics to build PharmGKB for FDA-approved breast cancer drugs [31]. Abdelhakim *et al*. created DDIEM for congenital defect drugs, covering treatments and phenotypes for 300 rare diseases, classifying treatments by mechanism, and speeding drug development for congenital diseases [32]. Kuo *et al*. developed RSDB, a rare skin disease database exploring new drugs through genes, compounds, and phenotype data [33]. Troulé *et al*. established DREIMT, a database associating drugs with immune features, prioritizing immunomodulatory drugs for cancer and autoimmune diseases [34]. Tao *et al*. constructed CancerHSP, an anti-cancer herb database with comprehensive data on herbs and components, aiding in understanding anti-cancer mechanisms across cancer cell lines [35]. Integrating public and patient data into disease-specific databases provides a comprehensive base for computational DR models.

Building on the concept that drugs with similar structures share similar functions, Eichborn *et al*. created the PROMISCUOUS database, linking drugs, proteins, and side effects *via* drug-target, drug-side effects, protein-protein, and drug-drug relationships. PROMISCUOUS predicts drug targets using structural similarities. For example, it associates the AD drug memantine's side effect of vomiting with its interaction with dopamine 2 receptors [36]. A decade later, Gallo *et al*. expanded this into PROMISCUOUS 2.0, enlarging its scope to include more entities and integrating drugs and targets with ICD-10 disease codes for disease-specific queries [37]. Huang *et al*. developed DMAP, a database detailing drug effects on genes or proteins, identifying gene expression profiles of candidate drugs to find new indications by comparison with FDA-approved drugs [38]. The emergence of online platforms like Pharmacorank, developed by Gnilopyat *et al*., has streamlined DR research, facilitating more comprehensive analysis and seamless data integration. Pharmacorank utilizes similarity analysis and data fusion to identify optimal drugs and targets [39].

Technological advancements have significantly lowered the costs associated with genomics research, making gene expression data indispensable in identifying disease similarities. The DNetDB database, developed by Yang *et al*., emphasized disease similarity through the exploration of gene regulation and analysis of gene expression to identify common dysfunctional mechanisms [40]. With genetic data supporting the majority of FDA approvals in 2021, Kang *et al*. developed PharmGWAS, a GWAS-based drug identification database, to explore disease-drug links through gene regulation [41]. Chen *et al*. introduced PharmOmics, analyzing drug effects and gene networks across species and tissues, aiding translational medicine and drug characterization [42]. As research evolves, the focus shifts from genetics to phenomics, with Xu *et al*. dRiskKB exploring disease phenotype similarities for new gene discoveries [43]. They also built a drug-side effect knowledge base from MEDLINE, enhancing DR research [44].

The variety of DR methods lacks standardized validation and systematic comparative evaluations. Brown *et al*. addressed this by creating repoDB, a database for assessing computational methods through approved and failed drugs [45]. Zhao *et al*. created EK-DRD, a database focused on experimental knowledge [46]. Tang *et al*. established the Drug Target Commons database, a network-based open platform enabling collaborative extraction, integration, annotation, and standardization by all users [47].

## 3 COMPUTATIONAL MODELS FOR DR

**Fig. (2):**
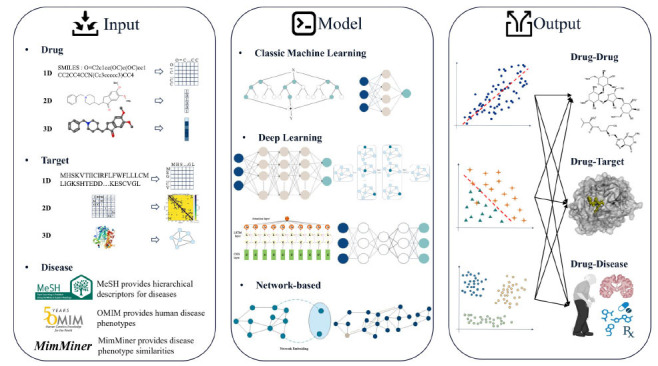
The input, model, and output sections of the DR computational model. DR models use representation learning to represent drugs as 1D SMILES, 2D fingerprints, and 3D graphs, and targets as 1D amino acid sequences, 2D matrix, and 3D graphs. Diseases are defined by phenotypic descriptions. DR model employs classic machine learning, deep learning, and network-based methods to find new drug uses, predict targets, and reveal drug-disease associations.

Data-driven computational models for DR mark a key innovation in precision medicine, enabling faster, more effective identification of new uses for existing drugs compared to traditional methods. These models integrate a wide array of heterogeneous biomedical data, including genomics, proteomics, clinical trials, and drug libraries. As computing and life sciences advance, DR models are evolving and showing significant potential. Effectively represent raw drug, target, and disease data through representation learning. They focus on three main inputs,*i.e*.: drug-drug, drug-target, and drug-disease association profiles, using classical machine learning (CML), deep learning (DL), and network-based methods (Fig. **2**).

### 3.1 Drug-drug Interactions Profiles Based DR Models

The DR model, centered on drug-drug interactions (DDI), analyzes the molecular structure, targets, and pharmacokinetics. Biomedical data constructs drug interaction networks, identifying shared targets and pathways (Table **2**).

Feng *et al*. introduced DPDDI, a network-based prediction model using Graph Convolutional Network (GCN) [48]. Yu *et al*. introduced the SumGNN model, leveraging a knowledge summarization Graph Neural Network (GNN) for predictions with explanatory reasoning paths [49]. Chen *et al*. introduced GCN-BMP, an end-to-end graph representation learning model, addressing feature reliance and noise [50]. Zhang *et al*. presented DDI-MDAE, a multi-modal deep autoencoder designed for handling large-scale and noisy datasets [51]. Huang *et al*. proposed skipGNN, aggregating direct and second-order interactions [52]. Zhao *et al*. introduced DDAGDL, a DL model emphasizing the non-Euclidean nature of biomedical network data. Utilizing an attention mechanism to mitigate data noise, it effectively learns drug and disease characteristics. The ensemble learning classifier XGBoost, within the framework of geometric DL, is employed to predict drug-drug associations in heterogeneous information networks [53]. Wang *et al*. proposed GCNMK, a multi-kernel graph convolutional network model, constructing a specific drug-drug interaction graph from two functional levels: activation and inhibition [54]. Fewer studies focus on drug-drug models due to the complexity of predicting new indications, requiring the integration of multidimensional data. Computational models must consider chemical genomics, target characteristics, and disease phenotypic information.

### 3.2 Drug-target Interactions Profiles Based DR Models

The goal of DR models is to enhance the understanding of the “gene-to-drug” process, centering on drug-target interactions (DTI) derived from gene expression. These models uncover complex interactions between existing drugs and potential targets, identifying new drug applications. Building these relationships involves analyzing direct drug-target interactions, molecular structures, biological functions, and roles in diseases. Research spans CML, DL, and network-based methods (Table **3**).

#### 3.2.1 Classical Machine Learning in DTI Prediction for DR

Early DR models used methods like Support Vector Machines and Decision Trees, focusing on feature learning from drug structures and target attributes. For example, Tong He's SimBoost model predicted drug-target affinity by constructing features from affinity similarities [55]. Multi-label classification models, like Yanyi Chu's DTI-MLCD, addressed one-to-many relationships between drugs and targets [56]. However, these models face challenges with high-dimensional data and dimensionality issues.

#### 3.2.2 Deep Learning in DTI Prediction for DR

DL's strong representational capabilities are increasingly applied to DR. Techniques like Convolutional Neural Networks (CNN), Recurrent Neural Networks (RNN), GNN, autoencoders, and Transformers extract meaningful features from complex data, offering new insights for DR. Wan *et al*. proposed NeoDTI, a nonlinear end-to-end learning model that integrates and extracts features of drugs and targets through neural networks, optimizing and predicting interaction in an end-to-end manner [57]. Peng *et al*. developed DTI-CNN, a deep neural network model based on feature representation learning. This model uses the Jaccard similarity coefficient to extract drug and target features, reducing dimensions with restarted random walks and denoising autoencoders to predict interaction in CNN [58]. However, CNNs have limitations in capturing contextual information, leading to a focus on NLP and GNN-based methods.

##### 3.2.2.1 NLP-based Models for DR

Natural Language Processing (NLP) methods enhance DR predictions. Öztürk *et al*. developed DeepDTA, using target amino acid sequences and drug SMILES strings in CNNs for prediction [59]. Karim Abbasi's DeepCDA added LSTM and attention mechanisms to this model, blending CNN and LSTM for encoding, enhancing generalizability with transfer learning [60]. Lesong Wei introduced MDL-CPI, employing BERT and CNN for target feature extraction and GNN for drug features [61]. Bomin Wei proposed DeepLPI, combining CNN and bidirectional LSTM in an end-to-end model [62]. Haixia Zhai's DGDTA model used dynamic GATs and bidirectional LSTM for DL-based predictions [63]. Ran Zhang's MHTAN-DTI applied Transformers and two-level attention to learning [64]. Mehdi Yazdani-Jahromi constructed the AttentionSiteDTI model, combining self-attention bidirectional LSTM mechanisms and graph embedding networks to merge structural features of small molecules and graph-represented protein binding sites, treating drug-target complexes as sentences [65]. Xinxing Yang's GraphCL-DTA is a graph contrastive learning model with molecular semantics [66]. In DR models, NLP faces challenges: drug and protein sequences' syntax varies from language, and methods might miss drugs' structures and proteins' properties. Converting sequences to sentences for NLP can result in losing vital information.

##### 3.2.2.2 GNN-based Models for DR

DL methods based on GNN have demonstrated superior capabilities in accurately representing the 3D structure of drugs and their complexes, extracting essential structural features, and providing interpretability. GNN-based methodologies include GCN, Graph Attention Networks (GAT), Graph Transition Networks (GTN), and Graph Isomorphism Networks (GIN). Current research predominantly revolves around exploring cross-domain method fusion based on GNNs to enhance overall model performance.

Jeongtae Son developed GraphBAR, a GCN-based model representing drug and target complexes as graphs of multiple adjacency matrices, saving time and resources compared to traditional CNNs [67]. Thin Nguyen introduced GraphDTA, representing drugs as graphs and predicting interaction using GCN, GAT, GIN, and GAT-GCN variants [68]. Jin Li proposed IMCHGAN, an inductive matrix completion with a heterogeneous GAT model for DTI prediction [69]. Yang Li developed DTI-MGNN, a multi-channel GCN and graph attention mechanism model, combining topological and semantic features to enhance learning [70]. Ziduo Yang introduced MGraphDTA, a deep multi-scale GNN model that analyzes models from a chemical perspective while capturing local and global drug structures [71]. Kanghao Shao proposed DTI-HETA, an end-to-end model with attention mechanisms in heterogeneous graphs, using GCN for embedding representation and an inner product decoder for prediction [72]. Mei Li presented MHGNN, a meta-path aggregated heterogeneous graph neural network, modeling complex entity relationships to capture high-order contextual dependencies [73]. Hongmei Wang developed DTI-GTN, a graph transformation network model using Jaccard similarity to generate feature vectors and predict interactions in a network graph [74]. Jiani Ma created MULGA, a unified multi-view learning and graph autoencoder framework, using multi-view learning to infer missing drug-target interactions [75]. However, shallow GNNs ignore global information and are highly dependent on graph attention mechanisms. Shang *et al*. introduced MccDTI, a multi-view network embedding model that combines deep autoencoders. This model predicts interaction through matrix completion, addressing the limitations of traditional GNNs, particularly in handling heterogeneous graphs and high-order dependency relationships [76]. Despite GNN's specialization in processing graph-structured data, current implementations are still limited by the available information for entity features.

In addition, a range of innovative models exploring novel concepts in DR. Yang *et al*. introduced the interpretability model ML-DTI, which incorporates a mutual learning layer between drug and target encoders. Notably, the mutual learning layer is predominantly implemented through multi-head attention and position-aware attention mechanisms [77]. Chu *et al*. proposed DTI-CDF, a cascade-based deep model designed to generate multiple similarity-based feature predictions for Drug-Target Interactions [78]. Tian *et al*. presented MHADTI, a prediction model that integrates a hierarchical attention mechanism and a multi-layer perceptron for enhanced performance [79].

#### 3.2.3 Network-based Models in DTI Prediction for DR

Network-based DR models integrate heterogeneous networks to represent diverse biological entities, extracting comparable information as features for drugs and targets. Luo *et al*. proposed DTINet, emphasizing compact feature learning. The model combines restart random walk network diffusion and dimensionality reduction through diffusion component analysis for low-dimensional vector representations [80]. Zhou *et al*. introduced MultiDTI, grounded in heterogeneous networks and multi-modal sequences, integrating similarity-based and network-based methods through joint multi-modal representation learning [81]. Peng *et al*. addressed drug substructures and targets with STD-ATC, a network-based inference model utilizing multiple fingerprint graphs [82]. Supervised contrastive learning proves effective for classifying nodes with identical labels. Li *et al*. proposed SGCL-DTI, an end-to-end supervised graph co-contrastive learning model. This model uses meta-path-guided graph encoders for low-dimensional representations, constructing topological and semantic graphs with drug-target pairs as nodes and training with contrastive loss [83]. Sameh K Mohamed introduced TriModel, a knowledge graph embedding model based on tensor decomposition, integrating data from four biomedical knowledge bases [84]. Ye *et al*. proposed KGE_NFM, a framework combining knowledge graphs and recommendation systems. After learning low-dimensional representations, this model integrates multimodal information using a neural factorization machine [85]. Overall, network-based models should consider node attributes, for they focus on constructing heterogeneous networks but may overlook intrinsic features of molecular types, posing challenges in leveraging potential knowledge for predictions.

### 3.3 Drug-disease Associations Profiles Based DR Models

Effective computational models for drug-disease associations (DDAs) on DR require a deep understanding of complex relationships among biomedical entities. This includes drugs, diseases, genes, miRNAs, signaling pathways, and cell lines. Incorporating genomics, miRNA, and cellular data into DR models enhances repositioning precision and propels personalized therapeutics (Table **4**).

#### 3.3.1 Classical Machine Learning in DDA Prediction for DR

In CML, with drug disease as input, Random Forest is widely used. Zhao *et al*. proposed HINGRL, a graph representation learning model predicting drug-drug interactions with a Random Forest classifier [86]. Xiaolu Xu introduced AutoBorutaRF, a feature selection model using an Autoencoder network and Boruta algorithm for prediction with a Random Forest classifier [87]. Zhang *et al*. presented RLFDDA, a meta-path-based graph representation learning model predicting outcomes with a Random Forest classifier [88].

#### 3.3.2 Deep Learning in DDA Prediction for DR

DL is effective in constructing heterogeneous networks with drug-disease side information. Notably, the GNN is widely used. Meng *et al*. proposed DRAGNN, a GNN model with weighted local information enhancement, using a graph attention mechanism for efficient node information collection, and prediction *via* a multi-layer perceptron [89]. Yu *et al*. introduced LAGCN, an end-to-end layer attention graph convolution network model, combining GCN for heterogeneous network information capture and a layer attention mechanism for embedding contributions [90]. Sun *et al*. presented PSGCN, a graph convolutional network model transforming interaction prediction into a graph classification task [91]. They also introduced AdaDR, an adaptive graph convolution network model extracting embeddings from node features and topological structures, learning adaptive weights through an attention mechanism [92]. Cai *et al*. proposed DRHGCN, an end-to-end model using a heterogeneous information fusion graph convolution network, enhancing prediction through layer attention mechanisms and inter/intra-domain feature extraction [93].

Other DL methods also explored DR. Zeng *et al*. developed deepDR, a network-based model using multi-modal deep autoencoders for high-level feature learning and prediction through multi-layer perceptron [94]. Liu *et al*. presented GLGMPNN, combining an optical gated message-passing neural network and a gated fusion mechanism [95]. Peng Chen *et al*. introduced DRDA, a model using deep autoencoders and adaptive fusion to reduce data sparseness impact and fuse multiple data sources [96]. Yi *et al*. proposed DDIPred, a deep gated recurrent unit model utilizing comprehensive similarity measures and Gaussian interaction profile kernel feature prediction through gated neural networks [97]. Jarada *et al*. proposed SNF-NN, a DL model based on similarity network fusion and neural network [98]. Liu *et al*. introduced HNet-DNN, a model based on deep neural networks extracting features in heterogeneous networks, using topological features for DNN training [99]. Akram Emdadi proposed Auto-HMM-LMF, a drug response prediction model based on autoencoders and hidden Markov models, selecting features and predicting drug response through logistic matrix decomposition [100]. Wang *et al*. introduced SRMF, a similar regularized matrix decomposition model predicting anti-cancer drug response based on gene expression profiles and drug structure in cell lines [101]. Chayaporn Suphavilai proposed CaDRReS, a model based on the recommendation system mapping drugs and cell lines to drug genomes for predictions [102]. Akram Emdadi proposed DSPLMF, a logistic matrix factorization model predicting drug cancer response [103]. Iwata *et al*. presented TT-WOPT, a tensor training weighted optimization model predicting drug-induced gene expression profiles in cell lines [104].

#### 3.3.3 Network-based Models in DDA Prediction for DR

Network-based methods reveal potential disease connections among biomedical entities. Meng *et al*. introduced DRWBNCF, a weighted bilinear neural collaborative filtering model utilizing nearest neighbors for noise reduction and improved accuracy [105]. Xie *et al*. proposed BGMSDDA, a bipartite graph diffusion model using multiple similarity integrals. They reconstructed the correlation matrix with the K-nearest neighbor algorithm, extracting features through linear domain and Gaussian kernel similarity [106]. Zhang *et al*. presented SCMFDD, a matrix factorization model based on drug feature and disease semantic similarity constraints [107]. Zhang *et al*. proposed DRIMC, using the Bayesian induction matrix and the similarity network fusion method for matrix reconstruction [108]. Yan *et al*. introduced MLMC, a multi-view learning prediction model based on matrix completion with Laplacian graph regularization for drug-disease associations [109]. Mengyun Yang proposed MSBMF, a multiple similarity bilinear matrix factorization model, and a prediction model using the multiplier alternating direction method [110]. Additionally, Yang *et al*. presented HGIMC, a matrix completion heterogeneous graph reasoning model filling missing entries with bounded matrix completion and enhancing drug-disease similarity through a Gaussian radial function for improved reasoning [111]. Ghorbanali *et al*. proposed DrugRep-HeSiaGraph, combining a drug-disease knowledge graph and a heterogeneous Siamese neural network. They constructed the DDKG-V1 knowledge graph and used HeSiaNet to predict drug-disease interactions [112]. Another knowledge graph, DrugRep-KG [113], was introduced. Yang *et al*. presented DRONet, combining network embedding and ranking learning, utilizing the effectiveness comparison between drugs for prioritization [114]. He *et al*. introduced HAMN, a hybrid attention memory network integrating two collaborative filtering models nonlinearly [115].

## 4 PRESENT STATUS DR FOR NDDS

The traditional “one drug, one target, one disease” approach falls short in addressing complex NDDs. “Network medicine” offers a systematic way to understand and treat NDDs [116]. DR models and platforms hold great potential in NDD research. The state-of-the-art computational methods and big data analysis provide a new approach for re-evaluating existing drugs with potential NDD applications.

### 4.1 NDDs Database for NDDs DR Model Development

NDD databases are crucial for constructing DR models, offering comprehensive data from basic research to clinical trials across various diseases. The development of databases and methods broadens the potential of applications on DR. The NDDs database categorically includes group diseases and individual diseases, with the former encompassing NDDs type and Polyglutamine (PolyQ) type, and the latter including AD, PD, HD, ALS, and Spinocerebellar ataxia (SCA) (Table **5**).

The NDDs type databases correspond to all relevant diseases. Xie *et al*. built the INDD database, mainly cataloging common and rare causes, clinical features, and pathological characteristics of NDDs such as AD, PD, ALS, and FTLD [117]. Vasaikar *et al*. developed NeuroDNet, an interactive database focusing on genes, signaling molecules, and proteins associated with 12 NDDs [118]. Na *et al*. established the first NDDs genetic modifications database, NeuroGeM, involving 9 genetic modifiers in *Drosophila melanogaster*, *C. elegans*, and *Saccharomyces cerevisiae* [119]. Yangyang *et al*. created the NDDVD database, cataloging genetic variant genes for 37 NDDs [120]. Chaudhari *et al*. introduced the hereditary ataxia data DINAX, focusing on genes and mutation information related to different hereditary ataxia subtypes [121]. Bi *et al*. built NDDRF, a database of NDD risk factors, aiming to advance personalized prevention, diagnosis, and treatment of NDDs [122].

PolyQ diseases, a subgroup of NDDs, share common characteristics. Szlachcic *et al*. curated the PolyQ database, gathering information on pathogenesis, disease phenotypes, and intervention measures for mouse models of polyglutamine diseases [123]. Estevam *et al*. developed the PolyQ Database, a disease-specific database for PolyQ diseases, encompassing epidemiological data, genes, proteins, disease physiology, and clinical characteristics [124].

#### 4.1.1 AD-related Databases

AD database is the most extensive of all disease-specific databases. Beekly *et al*. created the National Alzheimer's Coordinating Center database, aggregating patient information from 29 AD centers funded by the National Institute on Aging in the US [125]. Bertram *et al*. compiled AlzGene, focusing on AD-related genes and polymorphisms [126].

Liu *et al*. integrated AD-related chemical genome information into AlzPlatform [127]. Bai *et al*. devised AlzBase, a comprehensive database of AD gene dysregulation, targeting genes with varying dysregulation frequencies in AD [128]. Fang *et al*. created AlzhCPI, a compound-protein interaction prediction knowledge base for AD, providing functionalities in system pharmacology and DR [129]. Zhou *et al*. developed AlzGPS, an online tool integrating genetics, genomics, proteomics, metabolomics, and drug information for AD target and DR exploration [130]. Wang *et al*. introduced AlzRiskMR, a database assessing the association between modifiable risk factors and AD [131]. Lin *et al*. constructed AlzCode, integrating various AD omics data types to analyze disease gene priorities [132]. Bajic *et al*. established the knowledge base DES-Amyloidoses on human amyloid and its related disease associations, offering a comprehensive perspective for disease analysis [133]. Peng *et al*. developed ADmeth, focusing on AD-related DNA differential methylation [134].

#### 4.1.2 PD-related Databases

The PD database ranks as the second largest disease-specific database. Yang *et al*. established PDbase using substantia nigra expression sequencing tags. It integrates information from various public databases [135]. Taccioli *et al*. created the Parkinson's disease gene expression database (ParkDB), the first relational database for querying PD gene expression [136]. Lill *et al*. curated PDGene from genetic-related research, focusing on PD-related genes and polymorphisms [137]. Ziv Gan-Or *et al*. formed the Quebec Parkinson's Network (QPN) database for PD patients in Quebec, collecting patient-related clinical data and biological samples [138]. Pintado-Grima *et al*. designed aSynPEP-DB, a peptide library for the presynaptic protein αSyn, featuring biological peptides inhibiting αSyn aggregation to alleviate PD symptoms [139].

#### 4.1.3 ALS-related Databases

Some rare diseases are cataloged within relevant databases. In the field of ALS, Miller *et al*. created the ALS Patient Care Database to improve the quality of care for ALS patients [140]. Wroe *et al*. established the Amyotrophic Lateral Sclerosis Online Database (ALSOD), focusing on genetic, proteomic, and bioinformatic data of ALS patients, including information about the SOD1 gene for exploring links with ALS phenotypes [141].

#### 4.1.4 HD-related Databases

In the HD, Schultz *et al*. retrospective study analyzed the Enroll-HD database, a global research platform for HD [142]. Kalathur *et al*. introduced HDNetDB, a network-formatted database that primarily compiles gene and protein data [143]. Mears *et al*. addressed the gap between mouse models and population data by constructing the HDSheep database using multi-omics data from the transgenic HD sheep model [144].

#### 4.1.5 SCA-related Databases

In the realm of SCA disease, Liu *et al*. established the Spinocerebellar Ataxia Candidate Gene Database (SCA db), utilizing known SCA subtypes as positive controls and cataloging 3185 candidate genes for 17 types of SCA [145]. Faruq *et al*. developed the SCA-LSVD database to explore connections from the genome to phenotype, encompassing data on age, symptoms, and genetic information of Indian SCA patients at onset [146].

### 4.2 DR Models for NDDs

Computational models of DR in NDDs mainly focus on AD, PD, and ALS. Extensive research has been conducted on AD models, emphasizing CML and DL, while with limited exploration of network-based methods. PD, a common NDD, has received less attention in AI and CML. Despite limited data for ALS, a rare disease, relevant models have been developed (Table **6**). We list frequently used DR models in NDDs. The DRIAD model leads in AD drug screening, correlating genes with drug targets to find 15 FDA-approved drugs, including 5 JAK inhibitors [147]. Yue *et al*. PD model, using GWAS and gene expression, indicates estradiol's utility [148]. Fiscon *et al*. ALS model, *via* the SAveRUNNER algorithm, pinpoints modafinil's therapeutic potential [149]. In NDD research, general DR models, including deepDR [94] and DRHGCN [93], have been validated alongside disease-specific ones. Both models have been extensively cited and empirically tested for AD and PD. The deepDR model accurately predicted 14 of the top 20 drugs for AD and PD, supported by scientific literature, highlighting its effectiveness in identifying therapeutic candidates. Similarly, the DRHGCN model's top 10 predictions for both diseases are backed by research, indicating high predictive accuracy. The use of these models allows for more precise identification of potential NDD treatments, providing a scientific basis for developing new therapeutic approaches. This underscores the importance of both specialized and general models in advancing NDD treatment discoveries. Despite their utility, computational models for NDDs are fragmented and require a unified knowledge base. This database should include both general and disease-specific models to advance personalized recommendations and NDDs drug repositioning.

#### 4.2.1 DR Models for AD

In the realm of CML, Rodriguez *et al*. introduced the Drug Repurposing in AD (DRIAD) framework. This innovative approach quantifies the correlation between AD severity (Braak stage) and the molecular mechanisms encoded in a gene name list. Leveraging 80 FDA-approved drugs, predominantly kinase inhibitors, tested on differentiated human nerve cells. DRIAD analyzed the gene perturbation list and revealed the potential therapeutic relationships [147]. Understanding the comorbidity link between diabetes mellitus (DM) and AD, Ghiam *et al*. constructed a comprehensive network encompassing mRNA, miRNA, lncRNA, and drugs. Utilizing the random forest algorithm, the study explored gene expression changes within this network, identifying potential drugs to address the comorbidity. This approach provides a novel perspective on treating the crosstalk between DM and AD [150].

In the application of DL, Chyr *et al*. proposed the DOTA algorithm, a DL model based on multimodal networks. Addressing the intricate nonlinear structures in network medicine, DOTA employed Multimodal AutoEncoders (MAE) to fuse multiple drug networks. Leveraging the Wasserstein Autoencoder (WAE), DOTA extracted low-dimensional feature information to predict drug-AD association scores. This innovative approach shows its potential in discovering novel drugs for AD treatment [151].

Taking a systems pharmacology-oriented approach, Wu *et al*. introduced the MultiDCP model, a multi-task transfer learning strategy. This model comprehensively screened drug candidates in gene regulatory networks, surpassing traditional drug-drug and disease-disease similarity approaches. By integrating various omics data and employing a semi-supervised training method, MultiDCP demonstrated enhanced accuracy in predicting differential gene expression. This approach holds promise for identifying potential AD treatments [152].

In the domain of network-based methods, Nian *et al*. harnessed knowledge graphs to deepen the understanding of AD and refine drug treatment. Through literature mining, an AD-related knowledge graph was constructed, exploring compounds, drugs, and dietary supplements. Utilizing the PubMedBERT classifier and rule-based noise reduction methods, a comparative analysis of three graph embedding techniques-TransE, DistMult, and ComplEx-revealed that TransE outperforms others in prediction accuracy. The study highlighted the therapeutic potential of drugs like Amifostine and Acyclovir, as well as the preventive benefits of dietary fiber, tea, eggs, electrolytes, fruits, honey, rice, and coffee [153].

#### 4.2.2 DR Models for PD

Yue *et al*. proposed a systems pharmacology framework for PD using GWAS genetic and gene expression microarray data. Integrating gene co-expression modules with biomolecular interaction network analysis identified 12 top-scoring drugs, with 5 having reported potential and 6 showing developmental promise [148]. This method holds potential for polygenic disease drug exploration. Jacek Haneczok employed supervised machine learning to predict drugs enhancing PINK1 expression for PD treatment, identifying six potential candidates [154].

#### 4.2.3 DR Models for ALS

In ALS research, Fiscon *et al*. used a network similarity-based method to find potential drugs, quantifying gene-target proximity with SAveRUNNER. Candidate drugs demonstrated counteraction of gene expression perturbations caused by ALS [149]. Paik *et al*. introduced ClinDR, combining EMR and genomic data to construct a drug-disease network. Similarities calculated through the guilt-by-association method were validated through animal experiments [155]. Papikinos *et al*. extracted gene signatures from muscle biopsy specimens to find drugs for ALS. Cluster analysis of chemical similarity with FDA-approved ALS drugs supported their findings [156].

### 4.3 The Translation Informatics Tools for DR in NDDs

DR in NDD research is critical, and translational informatics tools are essential. These tools, including databases and predictive models, aid in drug discovery and repositioning.

DR starts with comprehensive databases. The AlzGPS platform (https://alzgps.lerner.ccf.org) showcases the value of multi-omics data in AD drug exploration. Users can search for drugs by name or ID to explore repositioning potential, like sildenafil and pioglitazone, which may reduce AD risk. The GENE section allows for gene-specific drug and omics data queries, like APOE [130]. The NDDRF (http://sysbio.org.cn/NDDRF/) is a key resource, that integrates data on lifestyle, epidemiology, and genetics. It has six modules for risk factor overview and specific queries. NDDRF is crucial for evaluating genes, targets, drugs, or diseases as positive or negative, enhancing predictive model assessment [122].

For predictive modeling in NDDs, deepDR (https://github.com/ChengF-Lab/deepDR) and DRHGCN (https://github.com/TheWall9/DRHGCN) are prominent. They use deep learning to link drugs and diseases across diverse data. deepDR uses autoencoders, while DRHGCN uses GCN and attention mechanisms. Both have been tested on AD and PD, with many predictions backed by clinical studies [93, 94]. Accessing these model repositories on GitHub allows us to download their source code and datasets, run them in our computational setups. Employing these predictive tools to forecast possible treatments for diseases provides key research insights.

Overall, translational informatics tools boost research efficiency and broaden drug discovery. Combining database analysis with model predictions helps identify potential drugs, crucial for developing new treatments and strategies. As technology advances, these tools will increasingly drive DR in NDD research.

## 5 FUTURE PERSPECTIVES ON DR FOR NDDS

For DR computational models, future efforts will prioritize method innovation, performance improvement, and integration with clinical practice. To improve the accuracy of the model, heterogeneous network integration methods must be optimized. This will involve comparing various models across diseases and approaches to find the best synergies. Creating reliable negative samples is vital for accurately reflecting drug-biomolecule non-interactions, improving model trustworthiness and applicability. A “gold standard” evaluation system is needed to validate the prediction credibility of a model. For DR platforms, developing personalized, disease-specific databases that interface with large language models like ChatGPT will advance precision medicine. Implementing real-time updates is key for swift access to the latest research and medical data. An open data-sharing platform will enhance collaborative efforts. In brief, computational advances and big data growth are driving DR towards major advancements, fostering innovative treatments for various diseases.

When applying DR for NDDs, common diseases like AD have rich data for integration and extraction, while rare ones like ALS lack sufficient data. Building and refining NDD databases is vital for deep disease comprehension. Interpretable Al models can clarify variable relationships of NDDs and their impact on treatment. Algorithms for drug combinations and network modeling can optimize multi-drug therapies. AI and online platforms foster a dependable and predictable research environment. These advancements form a strong foundation for future DR research against NDDs.

## CONCLUSION

In conclusion, translational informatics is crucial for DR research in NDDs, including combining data, models, and clinical insights for therapeutic innovation. Data richness is important for identifying drug candidates and targets, while AI models help to explore chemical space and identify drug efficacy. Research should enrich NDD databases for a comprehensive disease insight. State-of-the-art AI models predict drug-target interactions and uncover NDD mechanisms for effective treatments. Drug combination and network modeling reveal synergies and map interconnections, offering a comprehensive view. Specialized NDD databases, interpretable Al models, and algorithms accelerate DR research, refining therapeutic precision for improved patient outcomes. Overall, the application of DR for NDDs will be advanced through translational informatics.

## Figures and Tables

**Fig. (1):**
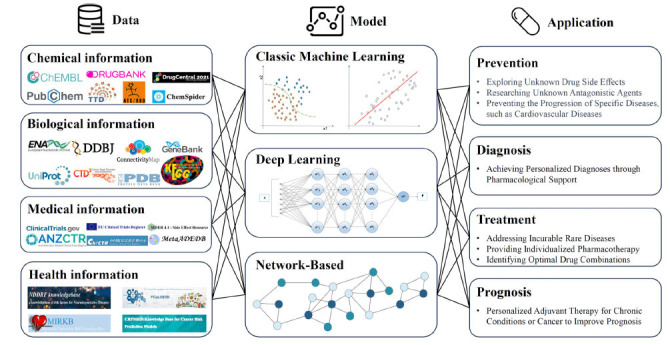
Translational informatics-driven drug repositioning research: data, model, and application. Drug repurposing models gather data from cheminformatics, bioinformatics, medical informatics, and health informatics, classified *via* translational informatics. They mainly employ classical machine learning, deep learning, and network-based to explore new drug applications for prevention, diagnosis, treatment, and prognosis. This pattern adds another layer to translational informatics, seamlessly merging data, models, and applications to aid the translation of research findings into clinical practice.

**Table 1:** Databases compilation for DR research.

**Database**	**Description**	**URL**	**References**
HCDT	HCDT includes 299,458 drugs, 5,618 genes, and 500,681 associations.	http://hainmu-biobigdata.com/hcdt	[24]
DrugMechDB	DrugMechDB has 4,583 drug indications for 1,580 drugs and 744 diseases, with a knowledge graph of 32,588 nodes and 32,249 edges.	https://sulab.github.io/DrugMechDB/	[25]
DrugR+	DrugR+ creats four datasets (enzymes, ion channels, GPCRs, nuclear receptors) and predicts drug-target interactions *via* machine learning.	http://www.drugr.ir	[26]
PharmGKB	PharmGKB is a pharmacogenetics knowledge base aids in understanding genetic impacts on drug responses.	https://www.pharmgkb.org/	[27]
DDIEM	DDIEM covers 300 rare diseases, 305 genes, and 584 drugs, linked to 1,482 phenotypes.	http://ddiem.phenomebrowser.net	[28]
RSDB	RSDB features 891 rare skin diseases, 28,077 genes, 9,732 phenotypes, 17,297 compounds, and extensive relationships.	https://rsdb.cmdm.tw36028515	[29]
DREIMT	DREIMT offers data on 4,960 drugs and 2,600 immune gene features.	http://www.dreimt.org	[30]
CancerHSP	CancerHSP features 2,439 anti-cancer herbal medicines with 3,575 components.	http://lsp.nwsuaf.edu.cn/CancerHSP.php	[31]
PROMISCUOUS	PROMISCUOUS is a network-based database featuring side effects, drug structures, protein interactions, and drug-protein interactions.	http://bioinformatics.charite.de/promiscuous	[32]
PROMISCUOUS2.0	PROMISCUOUS2.0 links new drugs, targets, and ICD-10 codes.	http://bioinformatics.charite.de/promiscously2	[33]
DMAP	DMAP lists 438,004 chemical-protein relationships across 24,121 compounds and 5,196 proteins.	http://bio.informatics.iupui.edu/cmaps	[34]
DNetDB	DNetDB records 108 diseases, 5,598 pathways, 7,357 genes, and 342 drug relationships, with 3,762 genes and 148 drugs common to multiple diseases.	http://app.scbit.org/DNetDB/	[35]
PharmGWAS	PharmGWAS collects Over 1,929 GWAS datasets and 724,485 features relate to 33,609 compounds.	https://ngdc.cncb.ac.cn/pharmgwas	[36]
PharmOmics	PharmOmics includes transcriptomic data from 20+ tissues with 13,530 samples (rats, humans, mice) and 941 drugs.	http://mergeomics.research.idre.ucla.edu/runpharmomics.php	[37]
dRiskKB	dRiskKB includes 34,448 disease risk pairs across 12,981 diseases.	http://nlp.case.edu/public/data/dRiskKB	[38]
repoDB	repoDB records 1,571 drugs and 2,051 diseases with 6,677 successful and 4,123 unsuccessful drug-indication pairs.	http://apps.chiragjpgroup.org/repoDB/	[39]
EK-DRD	EK-DRD annotates 1,861 FDA-approved and 102 withdrawn drugs with detailed assay records.	http://www.idruglab.com/drd/index.php	[40]
Drug Target Commons	Drug Target Commons offers an open platform for managing, annotating, and integrating drug target bioactivity data.	https://drugtargetcommons.fimm.fi/	[41]

**Table 2:** DDI models compilation for DR research.

**Model**	**Concepts and Thought**	**PMID**	**References**
DPDDI	A prediction model based on network information	32972364	[44]
SumGNN	A knowledge summarization graph neural network model	33769494	[45]
GCN-BMP	An end-to-end graph convolutional network model for graph representation learning	32622985	[46]
DDI-MDAE	A drug representation model based on a multimodal deep autoencoder	32497603	[47]
SkipGNN	A graph neural network model for predicting molecular interactions	33273494	[48]
DDAGDL	Utilizing geometric deep learning for handling heterogeneous networks with non-euclidean properties	36125202	[49]
GCNMK	A multi-kernel graph convolutional network model	29086119	[50]

**Table 3:** DTI models compilation for DR research.

**Model**	**Concepts and Thought**	**PMID**	**References**
SimBoost	Gradient-Boosted Regression Trees for Drug-Target Interaction Prediction	34864856	[51]
DTI-MLCD	Enhancing Multi-Label Classification with Community Detection	32964234	[52]
NeoDTI	A Nonlinear End-to-End Learning Model	30561548	[53]
DTI-CNN	DTI Prediction with Feature Extractors, Autoencoders and CNNs	32938374	[54]
DeepDTA	Integrating Fully Connected Layers in CNN for Sequence Learning	30423097	[55]
DeepCDA	Prediction Model with CNNs, LSTMs, Attention and Transfer Learning	32462178	[56]
MDL-CPI	Multi-View Deep Learning Model	35114401	[57]
DeepLPI	End-to-End Model composed of CNNs and Bidirectional LSTM Networks	36307509	[58]
DGDTA	Dynamic GATs and Bi-LSTM	37777712	[59]
MHTAN-DTI	Model with Meta-Paths, Transformers and Attention	36892155	[60]
AttentionSiteDTI	Integrates self-attention Bi-LSTMs and graph embeddings	35817396	[61]
GraphCL-DTA	A Graph Contrastive Learning Model with Molecular Semantics	38190664	[62]
GraphBAR	Protein-Target Binding Affinity Evaluation with Graph-Represented Atom Features	33831016	[63]
GraphDTA	GNN Model Representing Drugs in Graph Form	33119053	[64]
IMCHGAN	DTI Prediction with Inductive Matrix Completion and Heterogeneous Graph Attention	34115592	[65]
DTI-MGNN	Multi-Channel GCN and Graph Attention Mechanisms	34661237	[66]
MGraphDTA	Multi-Scale GNN and Gradient-Weighted Affinity Mapping for DTI Prediction	35173947	[67]
DTI-HETA	GCN, GAT and Inner Product Decoder	35380622	[68]
MHGNN	Efficient Heterogeneous Graph Learning with Metapath Modeling for DPI	36592060	[69]
DTI-GTN	Graph Transformation Network Model	36329406	[70]
MULGA	Unsupervised Molecular Similarity Analysis with Multi-View Learning and Autoencoders	37610353	[71]
MccDTI	Deep Autoencoders and Matrix Completion	35262678	[72]
ML-DTI	Bridging Drug-Target Encoders with Mutual Learning Layers	33904745	[73]
DTI-CDF	Cascade-Based Deep Model for Multi-Feature	31885041	[74]
MHADTI	DTI Prediction with Hierarchical Attention and Multi-Layer Perceptrons	36242566	[75]
DTINet	Multi-Kernel GCN Model	28924171	[76]
MultiDTI	Heterogeneous Network and Sequence Multimodal	34180970	[77]
STD-ATC	Model with Network Reasoning for Drug Substructures and Targets	32221522	[78]
SGCL-DTI	End-to-End Supervised Graph Collaborative Contrastive Learning for DTI	35561181	[79]
TriModel	Tensor Decomposition-Based KG Embedding	31368482	[80]
KGE_NFM	KG and Recommender System Integration	34811351	[81]

**Table 4:** DDA models compilation for DR research.

**Model**	**Concepts and Thought**	**PMID**	**References**
HINGRL	Graph representation learning model	34891172	[82]
AutoBorutaRF	Feature selection model	30972101	[83]
RLFDDA	Meta-path graph representation model	36456957	[84]
DRAGNN	Weighted local info enhancement GNN	38019732	[85]
LAGCN	End-to-end layer attention GCN Model	33078832	[86]
PSGCN	Graph convolutional end-to-end model	35921345	[87]
AdaDR	Adaptive graph convolution with attention	38070161	[88]
DRHGCN	Heterogeneous information fusion GCN model	34378011	[89]
deepDR	Multi-modal deep encoder and VAE model	31116390	[90]
GLGMPNN	Gated message passing and gating fusion model	36305457	[91]
DRDA	Deep autoencoder and adaptive fusion model	34717542	[92]
DDIPred	Deep gated recurrent unit model	34074242	[93]
SNF-NN	Similarity network fusion neural network	33482713	[94]
HNet-DNN	Deep feature extraction heterogeneous network	32118415	[95]
Auto-HMM-LMF	Drug response prediction with autoencoder and hidden markov model	33509079	[96]
SRMF	Regularized matrix factorization model	28768489	[97]
CaDRReS	Recommender system model	29868820	[98]
DSPLMF	Logic matrix factorization model	32174963	[99]
TT-WOPT	Tensor training weighted optimization model	31510663	[100]
DRWBNCF	Weighted bilinear neural CF model	35039838	[101]
BGMSDDA	Bipartite diffusion with similarity integration	34610633	[102]
SCMFDD	Drug-disease similarity constrained matrix factorization model	29914348	[102]
DRIMC	Bayesian inductive matrix completion model	31999326	[103]
MLMC	Multi-view matrix completion prediction model	35289352	[104]
MSBMF	Multi-similarity bilinear matrix factorization	33147616	[105]
HGIMC	Matrix completion heterogeneous graph inference model	33331887	[106]
DrugRep-HeSiaGraph	Model combining drug-disease knowledge graph and heterogeneous siamese neural networks	37789314	[108]
DrugRep-KG	A two-step approach combining drug-disease knowledge graphs with a heterogeneous Siamese neural network.	37023229	[107]
DRONet	Network embeddings and ranking earning	36562715	[108]
HAMN	Hybrid attention memory network model	33297947	[109]

**Table 5:** NDD databases for future DR.

**Diseases**	**Database**	**Potential Applications in NDDs DR**	**URL**	**PMID**	**References**
NDDs	INDD	Information on AD, PD, ALS and frontotemporal lobar degeneration.	https://www.gaaindata.org/partner/INDD	21784346	[113]
	NeuroDNet	Comprehensive gene, signaling molecule and protein info for 12 NDDs.	https://bioschool.iitd.ernet.in/NeuroDNet/	23286825	[114]
	NeuroGeM	Integrated data on 9 genetic modifiers.	https://neurogem.msl.ubc.ca/	24229347	[115]
	NDDVD	Information on 37 NDDs-associated genes and genetic variations.	http://www.sysbio.org.cn/NDDVD/genes	29688368	[116]
	DINAX	Data on hereditary ataxia genes and variations.	http://slsdb.manipal.edu/dinax	33007622	[117]
	NDDRF	Personalized NDDs prevention risk factors.	http://sysbio.org.cn/NDDRF/	36100329	[118]
PolyQ	PolyQ database	Mouse model data for polyQ disease family.	http://conyza.man.poznan.pl/	26515641	[119]
	PolyQ Database	Varied data on PolyQ.	https://polyq.pt/	37599593	[120]
AD	NACC	Clinical data for US AD patients.	https://naccdata.org/	15592144	[121]
	AlzGene	AD gene and polymorphism information.	http://www.alzgene.org/	17192785	[122]
	AlzPlatform	Chemical genomics data for AD genes, proteins, drugs.	https://www.cbligand.org/AD/	24597646	[123]
	AlzBase	AD gene dysregulation frequency.	http://alz.big.ac.cn/alzBase	25432889	[124]
	AlzhCPI	Compound-protein interaction predictions.	http://rcidm.org/AlzhCPI/index.html	28542505	[125]
	AlzGPS	Forecasts for AD targets.	https://alzgps.lerner.ccf.org/	33441136	[126]
	AlzRiskMR	Evaluation of modifiable risk factors for AD.	https://whve.gitbook.io/riskad/	32951050	[127]
	AlzCode	Functional genomics data for AD.	http://www.alzcode.xyz	35040932	[128]
	DES-Amyloidoses	Amyloid protein network relationships.	https://www.cbrc.kaust.edu.sa/des-amyloidosis/	35877764	[129]
	ADmeth	DNA methylation data for AD.	http://www.biobdlab.cn/ADmeth	35617175	[130]
PD	PDbase	PD gene data, variations, and functional elements.	http://bioportal.kobic.re.kr/PDbase/	19958497	[131]
	ParkDB	PD gene expression search.	http://www2.cancer.ucl.ac.uk/Parkinson_Db2/	21593080	[132]
	PDGene	Offer data on PD genes and polymorphisms.	http://www.pdgene.org/	22438815	[133]
	QPN	Canadian PD patient data.	https://rpq-qpn.ca/en/home/	31868683	[134]
	αSynPEP-DB	αSyn-regulating biopeptide traits.	https://asynpepdb.ppmclab.com/	38011719	[135]
ALS	ALS Patient Care Database	ALS patient care data for North America.	https://www.outcomes-umassmed.org/als/	10636125	[136]
	ALSOD	ALS genetic, proteomic, and bioinformatics analysis.	http://alsod.iop.kcl.ac.uk/als/	18608099	[137]
HD	Enroll-HD	Core data for HD patients.	https://www.enroll-hd.org/	28148631	[138]
	HDNetDB	HD gene annotation, associations and expression.	http://hdnetdb.sysbiolab.eu/	28701700	[139]
	HDSheep	Query specific genes, proteins and metabolites.	https://hdsheep.cer.auckland.ac.nz/	34420978	[140]
SCA	SCA db	SCA candidate gene data.	http://ymbc.ym.edu.tw/sca/	15217823	[141]
	SCA-LSVD	Indian SCA patient clinical characteristics.	http://miracle.igib.res.in/ataxia	19370769	[142]

**Table 6:** Existing DR models and their applications in NDDs.

**Diseases**	**Model Refernences**	**Description**	**Advantages**	**Disadvantages**	**Predictive Drugs**
AD	DRIAD [143]	Assess the link between Braak stages of AD severity and gene name encoded molecular mechanisms.	Enhance prediction accuracy by pre-model data filtering and gene set enrichment.	Study kinase inhibitors, ignoring drug blood-brain barrier permeability.	Ruxolitinib, Regorafenib
	[144]	Utilizing network and ML techniques to study DM and AD-related mRNA, miRNA, and lncRNA, and suggest drug treatments.	Investigate lncRNA's role in DM and AD medication interactions.	Validate drug negativity criteria.	Chromium, Nicotinate, Thiotepa
	DOTA [145]	Integrate drug networks with a multimodal autoencoder for feature extraction and prediction *via* Wasserstein encoding.	Construct a deep learning-based network for drug-target-adverse effect prediction.	False negatives may result from a limited sample size in negative instances.	Quetiapine, Aripiprazole, Risperidone, Suvorexant, Brexpiprazole, Olanzapine, Trazadone
	MultiDCP [146]	Forecast gene expression and cell viability under various drug concentrations.	Addresses the gaps in phenotype filtering.	The autoencoder's noise reduction is limited, relying only on genomic data.	Ibuprofen
PD	[147]	Investigating how drugs affect PD, using adverse reactions to understand these links.	Constructing a heterogeneous network tailored for PD	The performance of the method lacks sufficient evaluation.	28 drugs
	[148]	Using *in vitro* data and scaffold splitting to assess models and forecast new PINK1-targeting drugs in meta-ensemble.	Predicting drugs targeting PINK1 based on molecular characteristics	The dataset is limited in size.	Nitazoxanide, Imidazolidines, Trifluoromethyl, benzenes, Anilides, Nitriles, Stilbenes, Steroid esters
ALS	[149]	It is possible to quantify the association between disease-related genes and drug targets	SAveRUNNER is applied for the first time in the ALS.	The genetic data for ALS is incomplete.	Amoxapine, Clomipramine, Mianserin, Modafinil
	ClinDR [150]	Modeling and analysis based on EMR and genomic data	Integration of clinical and molecular features of both drugs and diseases	Analysis limited to a single EMR database	Tetrahydrozoline

## LIST OF ABBREVIATIONS

AD: Alzheimer's Disease
ALS: Amyotrophic Lateral Sclerosis
ALSOD: Amyotrophic Lateral Sclerosis Online Database
AI: Artificial Intelligence
CML: Classical Machine Learning
CNN: Convolutional Neural Networks
DDA: Drug Disease Associations
DDI: Drug Drug Interactions
DL: Deep Learning
DR: Drug Repositioning
DM: Diabetes Mellitus
DTI: Drug Target Interactions
GAT: Graph Attention Networks
GCN: Graph Convolutional Networks
GIN: Graph Isomorphism Networks
GNN: Graph Neural Networks
GTN: Graph Transition Networks
GWAS: Genome Wide Association Study
HD: Huntington's Disease
MAE: Multimodal Autoencoders
NLP: Natural Language Processing
NDDs: Neurodegenerative Diseases
PD: Parkinson's Disease
polyQ: Polyglutamine
QPN: Quebec Parkinson Network
RNN: Recurrent Neural Networks
SCA: SpinoCerebellar Ataxia
SCA db: Spinocerebellar Ataxia Candidate Gene Database
WAE: Wasserstein Autoencoder
